# Microbiological testing of pharmaceuticals and cosmetics in Egypt

**DOI:** 10.1186/s12866-015-0609-z

**Published:** 2015-12-09

**Authors:** Hend Zeitoun, Mervat Kassem, Dina Raafat, Hamida AbouShlieb, Nourhan Fanaki

**Affiliations:** Department of Pharmaceutical Microbiology, Faculty of Pharmacy, Alexandria University, Alexandria, 1 Khartoum Square, Azarita, Alexandria 21521 Egypt

**Keywords:** Pharmaceutical contaminants, Biochemical identification, Molecular identification, Artificial contamination, Minimum detection limit

## Abstract

**Background:**

Microbial contamination of pharmaceuticals poses a great problem to the pharmaceutical manufacturing process, especially from a medical as well as an economic point of view. Depending upon the product and its intended use, the identification of isolates should not merely be limited to the United States Pharmacopeia (USP) indicator organisms.

**Results:**

Eighty-five pre-used non-sterile pharmaceuticals collected from random consumers in Egypt were examined for the eventual presence of bacterial contaminants. Forty-one bacterial contaminants were isolated from 31 of the tested preparations. These isolates were subjected to biochemical identification by both conventional tests as well as API kits, which were sufficient for the accurate identification of only 11 out of the 41 bacterial contaminants (26.8 %) to the species level. The remaining isolates were inconclusively identified or showed contradictory results after using both biochemical methods. Using molecular methods, 24 isolates (58.5 %) were successfully identified to the species level. Moreover, polymerase chain reaction (PCR) assays were compared to standard biochemical methods in the detection of pharmacopoeial bacterial indicators in artificially-contaminated pharmaceutical samples.

**Conclusion:**

PCR-based methods proved to be superior regarding speed, cost-effectiveness and sensitivity. Therefore, pharmaceutical manufacturers would be advised to adopt PCR-based methods in the microbiological quality testing of pharmaceuticals in the future.

**Electronic supplementary material:**

The online version of this article (doi:10.1186/s12866-015-0609-z) contains supplementary material, which is available to authorized users.

## Background

Microbial contamination of pharmaceuticals has been an everlasting problem for researchers as well as pharmaceutical manufacturers worldwide. It can result in the spoilage of the formula by breaking down active ingredients and excipients, affecting the potency, stability and efficacy of the drug [1]. Furthermore, the presence of high numbers of pathogens poses a serious health threat to consumers, especially those who are already ill or in a weakened state [1–3]. Several cases of infection due to contaminated pharmaceuticals were reported in literature [4–6].

Common pharmaceutical contaminants include bacteria, where contamination with Gram-positive bacteria implicates human intervention as a major reason for product contamination, while the presence of Gram-negative bacteria suggests lack of process control in pharmaceutical environments, especially involving water systems and raw materials [4]. Gram-negative rods are considered the most commonly found bacterial isolates in non-sterile pharmaceuticals, regardless of geographical location or time [4, 7].

Conventional standard microbiological methods are currently used for the routine testing of pharmaceutical products and identification of pharmaceutical bacterial contaminants, including conventional pharmacopoeial methods, as well as simplified, commercially available biochemical test kits, such as the API system [8, 9]. Recently, however, molecular technologies have positively affected the field of pharmaceutical microbiology, providing rapid quantitative as well as qualitative information on microorganisms present in a given pharmaceutical sample [10–12]. The *16S rRNA* gene is most commonly used for taxonomic purposes [13, 14].

The objective of this study was to identify bacterial contaminants, isolated from different pharmaceuticals using conventional biochemical methods and the API identification system. Bacterial isolates showing inconclusive or contradictory results were further subjected to polymerase chain reaction (PCR)-based methods of identification using either universal or species-specific primer pairs.

Moreover, this study aimed at comparing between standard biochemical methods and PCR-based assays in the detection of pharmacopoeial bacterial indicators in artificially-contaminated pharmaceutical samples with respect to time, cost and limit of detection.

## Methods

### Standard bacterial strains

The following standard strains were used: *Staphylococcus aureus* ATCC 6538P*, Staphylococcus epidermidis* ATCC 12228, *Bacillus subtilis* ATCC 6633, *Bacillus cereus* ATCC 14579, *Escherichia coli* NCTC 10418*, Klebsiella pneumoniae* ATCC 13883*, Salmonella enterica subspecies enterica* ATCC 14028, and *Pseudomonas aeruginosa* ATCC 9027.

### Tested pharmaceuticals

A total of 85 used pharmaceuticals (taken after verbal consent from the consumers) were tested for bacterial contamination. These included 28 pharmaceutical preparations, 33 cosmetic preparations, 17 raw materials and 7 herbal products.

### Isolation of contaminants

One gram (or 10 ml) of the tested pharmaceutical were aseptically added to 20 ml of sterile tryptic soy broth (TSB) with or without PLT (polysorbate-20 5 % v/v, lecithin 0.3 % w/v, thioglycolate 0.01 % w/v) as a neutralizing agent [15] and incubated at 37 °C for 24–48 h in a shaking incubator at 110 rpm. Fifty microliters (μl) aliquots of the overnight cultures were then streaked onto nutrient agar plates and incubated at 37 °C for 24 h.

### Identification of the bacterial isolates by biochemical tests

After Gram-staining, the bacterial isolates were identified according to the “Identification flow charts” of Bergey’s Manual of Determinative Bacteriology [16].

The Gram-positive cocci were streaked onto the surface of mannitol salt agar (MSA), and then identified by subjecting them to confirmatory tests specific for *Staphylococcus* spp., including catalase test, tube coagulase test, DNase test, blood hemolysis, urease test and novobiocin sensitivity test. The tested isolates were further identified using the API Staph kit (BioMérieux, France) according to the manufacturer’s instructions. Sporulating, rod-shaped Gram-positive isolates were subjected to spore staining to confirm the presence of *Bacillus* spp. They were subsequently subjected to biochemical tests, namely starch hydrolysis (test for amylase), Voges Proskauer and catalase tests [16–19].

On the other hand, Gram-negative isolates were respectively streaked onto the surface of both MacConkey’s agar and cetrimide agar. Oxidase-negative Gram-negative isolates, growing only on MacConkey’s agar (enterobacteria) were further identified using various biochemical tests including indole production, methyl red, Voges Proskauer, citrate utilization, urease, oxidative/fermentative activity, motility tests as well as growth on triple sugar iron agar and xylose lysine desoxycholate agar [17, 20, 21]. Further biochemical identification of Gram-negative isolates was done using the API 20E kit (BioMérieux, France).

### PCR-based identification

#### Identification using species-specific primer pairs

PCR-based identification of a specific bacterial species was carried out using a DNA extraction protocol [22], and the species-specific primer pairs were designed using the Standard Nucleotide Basic Local Alignment Search Tool (BLAST) [23]. The adopted species-specific primer pairs and universal primer pairs are listed in Table 1.

**Table 1:** PCR primers used in this study

Bacterial target/Primer name	Forward (F) and Reverse (R) primer Annealing temperature (tm-5 °C)	Nucleo-tide positions^a^	Gene amplified	PCR product size (bp)	Ref.
Universal primer pair	F: 5′AGAGTTTGATCMTGGCTCAG3′	NP	*16S rRNA*	1500	[27]
	R: 5′TACGGYACCTTGTTACGACTT3′				
	(46.8 °C)				
*S. hominis*	F: 5′GTTCGATAGTGAAAGATGGCTC3′	NP	*16S rRNA*	833-852	[24]
	R: 5′GGAAACTTCTATCTCTAGAAGG3′				
	(43.4 °C)				
*S. warneri*	F: 5′GGTTCAATAGTGAAAGGCGGC3′	NP	*16S rRNA*	833-852	[24]
	R: 5′GGAAGACTCTATCTCTAGAGC3′				
	(41.1 °C)				
*S. epidermidis*	F: 5′TCTCTTTTAATTTCATCTTCAATTCCATAG3′	448-477	^b^	174	[25]
	R: 5′AAACACAATTACAGTCTGTTATCCATATC3′	593-622			
	(54 °C)				
*B. anthracis*	F: 5′AATCGTAATATTAAACTGACG3′	607-627	*gyrB*	244	[26]
	R: 5′CCTTCATACGTGTGAATGTTG3′	831-851			
	(40.5 °C)				
*B. cereus*	F: 5′ATTGGTGACACCGATCAAACA3′	490-510	*gyrB*	364	[26]
	R: 5′TCATACGTATGGATGTTATTC3′	834 -854			
	(41 °C)				
*B. subtilis*	F:5′CAGTCAGGAAATGCGTACGTC CTT3′	NP	*gyrA*	1027	[26]
	R:5′CAAGGTAATGCTCCAGGCATTGCT3′				
	(57.2 °C)				
*S. aureus*	F: 5′GCGATTGATGGTGATACGGTT3′	48-70	*nucA* (nuclease A)	280	[32]
	R: 5′AGCCAAGCCTTGACGAACTAA AGC3′	303-328			
	(55 °C)				
*E. coli*	F: 5′AAAACGGCAAGAAAAAGCAG3′	754-773	*uidA* (*β*-D-glucuro-nidase)	147	[31]
	R: 5′ACGCGTGGTTACAGTCTTGCG3′	880-900			
	(50.7 °C)				
*S. enterica*	F: 5′ATCGCCACGTTCGGGCAATTC3′	NP	*invA* (invasion protein)	275	[33]
	R: 5′ACGGTTCCTTTGACGGTGCGAT3′				
	(55 °C)				
*P. aeruginosa*	F: 5′ATGGAAATGCTGAAATTCGGC3′	NP	*oprL* (membrane lipoprotein)	504	[28, 29]
	R: 5′CTTCTTCAGCTCGACGCGACG3′				
	(55 °C)				

^a^Nucleotide positions: refers to the positions of the nucleotides on the target gene where the forward and reverse primers anneal

^b^A genomic DNA fragment with unknown coding potential

*NP* Not Provided

Six species-specific primer pairs for *S. hominis, S. warneri* [24]*, S. epidermidis* [25]*, B. subtilis, B. cereus* and *B. anthracis* [26] were used for further identification of selected staphylococcal and *Bacillus* isolates, respectively, by a simple uniplex PCR-based method. Appropriate positive and negative controls were included to exclude false negative and false positive results [27]. The primers for *S. epidermidis* ATCC 12228, *S. warneri* isolate (36), *B. subtilis* ATCC 6633 and *B. cereus* ATCC 14579 were used as the positive controls for their respective strains, whereas the *16S rRNA* universal primer pair was used as a positive control for *S. hominis* and *B. anthracis.* On the other hand, *S. aureus* ATCC 6538P was used as the negative control for *S. epidermidis****,****S. warneri* and *S. hominis*. In addition, *B. cereus* ATCC 14579 was used as a negative control for *B. subtilis* while *B. subtilis* ATCC 6633 acted as a negative control for *B. cereus* and *B. anthracis*.

The protocol of amplification for all of the tested bacterial species was as follows: 12.5 μl of 2× Green PCR master mix (DreamTaq™, Fermentas Life Sciences, France), 5 μl DNA template (10 pg-1 μg), 0.4 μM of each primer, and 5.5 μl PCR-grade water.

The PCR reactions were performed using a Perkin Elmer thermocycler (Gene Amp PCR system 2400, USA), with an initial denaturation at 95 °C for 5 min, followed by 30 cycles at 95°C (0.5 min), Tm-5 (0.5 min) and 72 °C (0.5-1.5 min) and a final extension at 72 °C for 10 min. The extension time during the 30 cycles was chosen based on the product size (1 min/kb).

The amplified samples were then analyzed by agarose gel electrophoresis using 1–1.5 % agarose gels (Fischer Scientific®, UK) that had been prestained with ethidium bromide (Fischer Scientific, Canada).

#### Identification using *16S rRNA* universal primer pair

Identification of selected bacterial isolates was done by PCR amplification of the*16S rRNA* gene using universal primer pair [28, 29], followed by *16S rRNA* gene sequencing of the amplified PCR products. Genomic bacterial DNA isolation kit (Biospin Bacteria Genomic DNA Extraction Kit, Cat# BSC12S1, China) was used to extract highly pure DNA, which was used for downstream molecular experiments, including DNA sequencing. Each PCR reaction tube (final volume, 50 μl) contained the following: 25 μl of 2× Green PCR master mix (DreamTaq™, Fermentas Life Sciences, France), 7 μl DNA extract (10 pg-1 μg), 0.6 μΜ of each primer, and 12 μl PCR-grade water. A negative control was prepared by replacing the DNA template with sterile deionized water (DiW). The PCR reaction was performed with an initial denaturation at 95 °C for 5 min, followed by 30 cycles at 95 °C, 46.8 °C and 72 °C for 0.5 min, 0.5 min and 1.5 min, respectively and a final extension at 72 °C for 10 min. The final PCR product was purified by the company Sigma for Scientific Services (Cairo, Egypt) prior to DNA sequencing of the *16S rRNA* gene with the help of a Bioneer automated sequencer (Bioneer 3730xl, USA), using forward and reverse *16S rRNA* universal primer pairs [28, 29].

### Sequence alignments and construction of phylogenetic trees

Using the Nucleotide Basic Local Alignment Search Tool (BLAST) [30], the nucleotide sequences were compared against the GenBank database. The top twenty homology matched hits for each isolate, with the highest total score, were then used together with the isolate sequence to construct a phylogenetic tree using the BLAST program.

### Artificial contamination of a pharmaceutical sample

Ten-gram samples of raw material glucose powder were added to 100 ml of TSB. After thorough dissolution, samples were inoculated separately with 1 ml of a 10^−6^ dilution of 24-h TSB cultures of either *P. aeruginosa* ATCC 9027, *E. coli* NCTC 10418, *S. aureus* ATCC 6538P or *S. enterica subsp. enterica* ATCC 14028. Inoculated samples were placed in a shaking incubator for 24 h at 37 °C and 100 rpm.

### Standard methods for isolation and detection of bacterial contaminants

After incubation, the enriched TSB cultures were streaked on MacConkey’s agar, MSA, cetrimide agar and Xylose-Lysine-Desoxycholate (XLD) agar plates. After incubation at 37 °C for 24–48 h, colonies were streaked onto sterile plates of trypticase soy agar (TSA) for isolation of pure cultures. TSA plates were incubated for 18–24 h and cells from pure cultures were Gram-stained and then subjected to biochemical identification tests. Additionally, API 20E and API Staph kits were used for further biochemical identification.

### DNA extraction from artificially-contaminated samples

Three ml-aliquots of the artificially-contaminated samples were centrifuged for 10 min at 14000 rpm. The pellet was washed twice in 1 ml of sterile DiW, then resuspended in 1 ml of sterile DiW and boiled for 10–15 min followed by centrifugation at 14000 rpm for 5 min. The supernatant was used as a source of DNA for the PCR reaction.

### PCR detection of bacterial contaminants

For each sample, 10 μl of the DNA lysate (10 pg-1 μg) were transferred into a PCR tube containing 12.5 μl 2× Green PCR master mix (DreamTaq™, Fermentas Life Sciences, France), 0.4 μM of each primer (Table 1), and 0.5 μl PCR-grade water. The simple uniplex PCR reaction conditions were optimized for the different species-specific primer pairs for each bacterial contaminant [28, 29, 31–33], so that they could be used in a single PCR run with an initial denaturation at 95 °C for 5 min, followed by 40 cycles at 95 °C, 55 °C and 72 °C for 0.5 min, 0.5 min and 0.75 min, respectively, and a final extension at 72 °C for 10 min.

### Determination of Minimum Detection limit (MDL) for both methods under investigation

The MDL of each method was determined by using standard *E. coli* NCTC 10418 as a representative of bacterial contaminants. The pharmaceutical product used was 10 % glucose solution prepared aseptically by adding 10 gm glucose powder to 100 ml sterile distilled water.

A TSB culture of *E. coli* NCTC 10418 was diluted in 0.9 % sterile saline to prepare a series of 10-fold dilutions. The 10 % glucose solutions were separately inoculated with the above mentioned dilutions to contain about 10^7^ - 10^2^ CFU/ml for each of the tested methods. To determine the MDL for conventional biochemical methods; a loopful of each inoculated sample was streaked onto selective media (MacConkey’s agar and XLD agar) as well as TSA plates. The MDL was estimated based on the least inoculum (CFU/ml of the sample) showing growth on agar plates.

To determine the MDL for PCR-based methods, the following was done: 1ml of each inoculated sample was centrifuged at 14000 rpm for 10 min. The supernatant was discarded and the pellet was resuspended in 5 μl sterile DiW which was then transferred into a PCR tube that was then heated at 95 °C for 10 min to extract DNA. The remaining PCR components were then added to the PCR tube: 12.5 μl of 2× Green PCR master mix (DreamTaq™, Fermentas Life Sciences, France), 0.4 μM of each primer, and 5.5 μl PCR-grade water.

Using the species-specific primer pair for *E. coli* [29, 31], the reaction was performed with an initial denaturation at 95 °C for 5 min, followed by 30 cycles at 95°C, 50.7°C and 72°C for 0.5 min each, and a final extension at 72°C for 10 min.

## Results

A total of 85 pre-used pharmaceuticals (Additional file 1) were collected and examined for the eventual presence of bacterial contaminants. The samples were collected from different available classes of preparations including syrups, elixirs, drops (eye, ear and oral), powders, teabags and cosmetics. In total, 41 bacterial contaminants were successfully isolated from 31 of the tested pharmaceuticals, i.e., bacterial contaminants were found in 36.5% of the tested products. These contaminants were distributed as follows: 9 from 28 pharmaceutical preparations, 10 from 17 raw materials, 12 from 33 cosmetic preparations and 10 from 7 herbal products. Gram-positive bacteria were more prevalent than Gram-negative bacteria in the tested products, where they constituted 88.9% (8/9), 91.7% (11/12) and 100% (10/10) of the detected contaminants in pharmaceutical preparations, cosmetic preparations and raw materials, respectively. On the other hand, 9 out of the 10 contaminants in herbal products were Gram-negative bacteria.

Following their isolation, the bacterial contaminants were subjected to biochemical identification, using conventional tests as well as API identification systems. Selected isolates, which showed inconclusive or contradictory results, were further identified using PCR-based methods.

### Biochemical identification of the bacterial isolates

Among the isolated contaminants, 30 isolates were Gram-positive (12 coagulase-negative staphylococci, 16 *Bacillus* spp., one *Kocuria*/*Micrococcus* spp. and one Gram-positive non-spore-forming rod), whereas the remaining 11 isolates were thin short Gram-negative rods (10 enterobacterial isolates and one *Pseudomonas* spp.).

Based on the biochemical identification results of the 13 Gram-positive cocci (Table 2), six isolates were accurately identified by both conventional methods and the API Staph system. On the other hand, the remaining 7 isolates and the Gram-positive, non-spore-forming, rod-shaped isolate 24B were not conclusively identified, and were thus further subjected to PCR-based methods to achieve proper identification.

**Table 2:** Identification of Gram-positive cocci using conventional biochemical methods and API Staph kit

Isolate code	Identification based on conventional biochemical tests	Identification based on API Staph kit		Final conclusion
		Result (%ID)	Level of discrimination	
16A	*S. epidermidis*	*S. epidermidis* (95.3 %)	Good	*S. epidermidis* ^a^
16B	*S. hominis, S. cohnii, S. saprophyticus, S. xylosus, S. simulans* or *S. warneri*	*S. saprophyticus* (64.6 %)	Not valid	^b^
17		*S. warneri* (61.5 %), *S. hominis* (35.5 %)	Good to genus	*S. warneri* or *S. hominis* ^c^
20A	*S. epidermidis*	*S. auricularis* (83.9 %)	Good to genus	^d^
20B	*S. epidermidis*	*S. epidermidis* (96.1 %)	Good	*S. epidermidis* ^a^
22	*S. epidermidis*	*S. hominis* (46 %), *S. warneri* (21.3 %)	Not valid	^b^
23	*S. haemolyticus*	*S. haemolyticus* (88.9 %)	Acceptable	*S. haemolyticus* ^a^
24A	Micrococcus spp.	*Kocuria varians/rosea* (97.8 %)	Good	*Kocuria varians* ^a^
36	*S. hominis, S. cohnii, S. saprophyticus, S. xylosus, S. simulans* or *S. warneri*	*S. warneri* (89.9 %)	Good to genus	*S. warneri* ^a^
44		*S. warneri* (61.5 %), *S. hominis* (35.5 %)	Good to genus	*S. warneri* or *S. hominis* ^c^
52	*S. epidermidis*	*S. chromogenes* (72.2 %)	Good to genus	^d^
57A	*S. epidermidis*	*S. epidermidis* (98.1 %)	Good	*S. epidermidis* ^*a*^
62	*S. hominis, S. cohnii, S. saprophyticus, S. xylosus, S. simulans* or *S. warneri*	*S. warneri* (61.5 %), *S. hominis* (35.5 %)	Good to genus	*S. warneri* or *S. hominis* ^c^

^a^Molecular identification was not deemed necessary

^b^The isolate was not properly identified by biochemical methods and therefore molecular identification was required

^c^Further identification to the exact Staphylococcus sp. using molecular methods was required

^d^Contradictory identification results using biochemical methods necessitate the use of molecular identification

The *Bacillus* contaminants, representing 39% of the isolated contaminants, were isolated from the tested raw materials, and their identity was confirmed by differential biochemical tests, including starch hydrolysis, Voges Proskauer and catalase tests (Additional file 2).

Based on the biochemical identification results of the 11 Gram-negative isolates (Table 3), only five were conclusively identified using both conventional methods and the API 20E system.

**Table 3:** Identification of Gram-negative isolates using biochemical methods

Isolate code	Identification based on conventional biochemical tests	Identification based on API 20E kit		Final conclusion
		Result (%ID)	Level of discrimination	
1	*Erwinia cacticida* or *Erwinia dissolvens*	*Ent. cloacae* (94.3 %)	Good	Contradiction (*Enterobacter/Erwinia* spp.)^a^
8A	*Providencia alcalifaciens, Providencia rustigianii* or *Providencia stuartii*	*Providencia alcalifaciens/rustigianii* (72 %)	Very good identification to the genus	*Providencia alcalifaciens/rustigianii* ^a^
11A	*Ent. aerogenes* or *K. pneumoniae subsp. pneumoniae*	*K. pneumoniae subsp. pneumoniae* (97.6 %)	Good	*K. pneumoniae subsp. pneumoniae* ^a^
11B	*Erwinia cacticida*	*Buttiauxella agrestis* (63 %)	Low	^b^
12	*Erwinia cacticida* or *Erwinia dissolvens*	*Ent. sakazakii* (51.1 %), *Ent. amnigenus 1* (31.7 %)	Excellent identification to the genus	Contradiction (*Enterobacter/Erwinia* spp.)^a^
54A	*Ent. cloacae* or *Ent. agglomerans*	*K. pneumoniae subsp. pneumoniae* (98 %)	Good	^c^
54B	*K. oxytoca*	*Ent. sakazakii* (99.9 %)	Excellent	^c^
55A	*Erwinia cacticida* or *Serratia entomophila*	*Pantoea* spp. 2 (50.7 %), *K. pneumoniae subsp. pneumoniae* (30.4 %)	Low	^b^
55B	*Ent. amnigenus, Ent. sakazakii* or *Ent. nimipressuralis*	*Ent. sakazakii* (98.4 %)	Good	*Ent. sakazakii* ^a^
56	*K. oxytoca*	*Ent. sakazakii* (99.9 %)	Excellent	^c^
82A	*Pseudomonas* spp. other than *P. aeruginosa*	Non fermentor spp. (32.1 %), *Ochrobactrum anthropi* (25.4 %), *Bordetella/ Alcaligenes/ Moraxella* spp. (24.4 %)	Low	^b^

^a^Molecular identification was not deemed necessary

^b^The isolate was not properly identified by biochemical methods and therefore molecular identification was required

^c^Contradictory identification results using biochemical methods necessitate the use of molecular identification

### Molecular identification using PCR-based methods

**Fig. 1:**
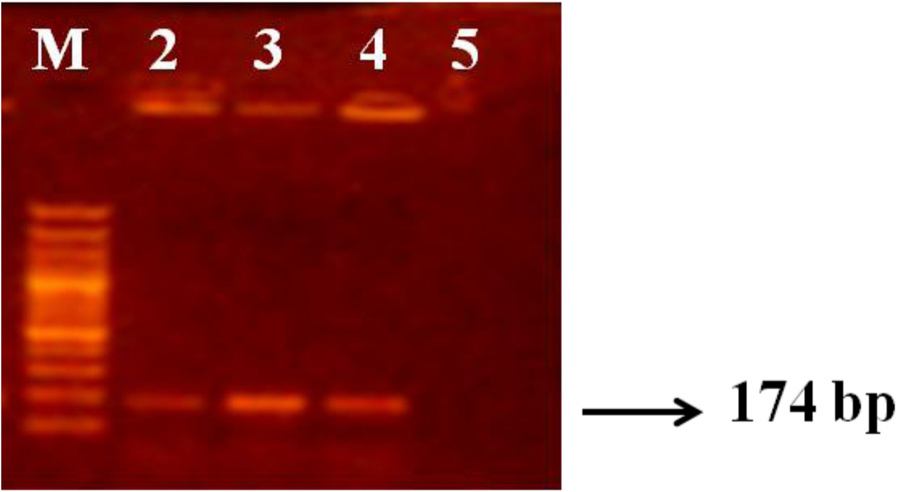
PCR amplification of specific gene fragment (174 bp) in *S. epidermidis.* Lane M: 100 bp plus DNA ladder*; Lane 2: isolate 20A; Lane 3: isolate 52; Lane 4: positive control (*S. epidermidis*ATCC 12228); Lane 5: negative control (*S. aureus*ATCC 6538P). * DNA ladder yields 14 fragments of the following sizes (bp; from top to bottom): 3000, 2000, 1500, 1200, **1000**, 900, 800, 700, 600, **500**, 400, 300, 200 and 100

Species-specific primer pairs were used whenever the results of the conventional and the API methods were contradictory, or when further identification to the species level was required. This pertained to 5 staphylococcal isolates (17, 20A, 44, 52 and 62; Table 1), as well as all of the *Bacillus* isolates. Two of the former isolates (20A and 52) showed a PCR product at the specified band size (174 bp) (Fig. 1) and were thus identified as *S. epidermidis*. The results of the PCR-based identification of these 2 isolates were in agreement with those of conventional biochemical tests, while they contradicted those of the API Staph kit.

The remaining 3 staphylococci (isolates 17, 44 and 62) were not definitively identified by biochemical methods (Table 2), though the API Staph kit results were more useful in guiding us towards choosing the specific primers (*S. warneri* and *S. hominis*-specific primers) used in the PCR-based identification. The 3 isolates were identified as *S. warneri*, since they showed a PCR product at the expected band size (about 850 bp) upon using the respective species-specific primer pair (Additional file 3).

For further identification of all of the 16 *Bacillus* isolates, three species-specific primer pairs were used, each was respectively specific to one of the 3 most pathogenic *Bacillus* spp.; *B. anthracis*, *B. subtilis* and *B. cereus*. None of the *Bacillus* isolates was identified as *B. anthracis*, while twelve isolates yielded a PCR product at the specified band size (1027 bp) after PCR amplification using a *B. subtilis*-specific primer pair (Additional files 4 and 5). It is worth mentioning that, although isolate 8B failed to hydrolyse starch when grown on starch agar and isolate 29 showed a negative Voges Proskauer test, they were both still identified as *B. subtilis* using the respective species-specific primer pair. Moreover, only one *Bacillus* isolate (isolate 13) turned out to be *B. cereus*, since it showed a PCR product at 364 bp upon using the respective species-specific primer pair (Table 1). As for the remaining three *Bacillus* isolates, none of them showed a PCR product with any of the three used primer pairs. Consequently, the latter isolates could not be identified by the applied PCR-based method.

The presence of three Gram-negative isolates showing contradictory results between both conventional tests and API 20E kit at the genus level (isolates 54A, 54B and 56; Table 3) necessitated the use of a PCR-based method using *Enterobacter*-specific and *Klebsiella*-specific primer pairs. However, this was found to be inapplicable, since the two genera share very close genotypic characteristics [34], which was confirmed by checking the specificity of the respective genus-specific primer pairs published in the literature [35–37] and using the BLAST program [23].

**Fig. 2:**
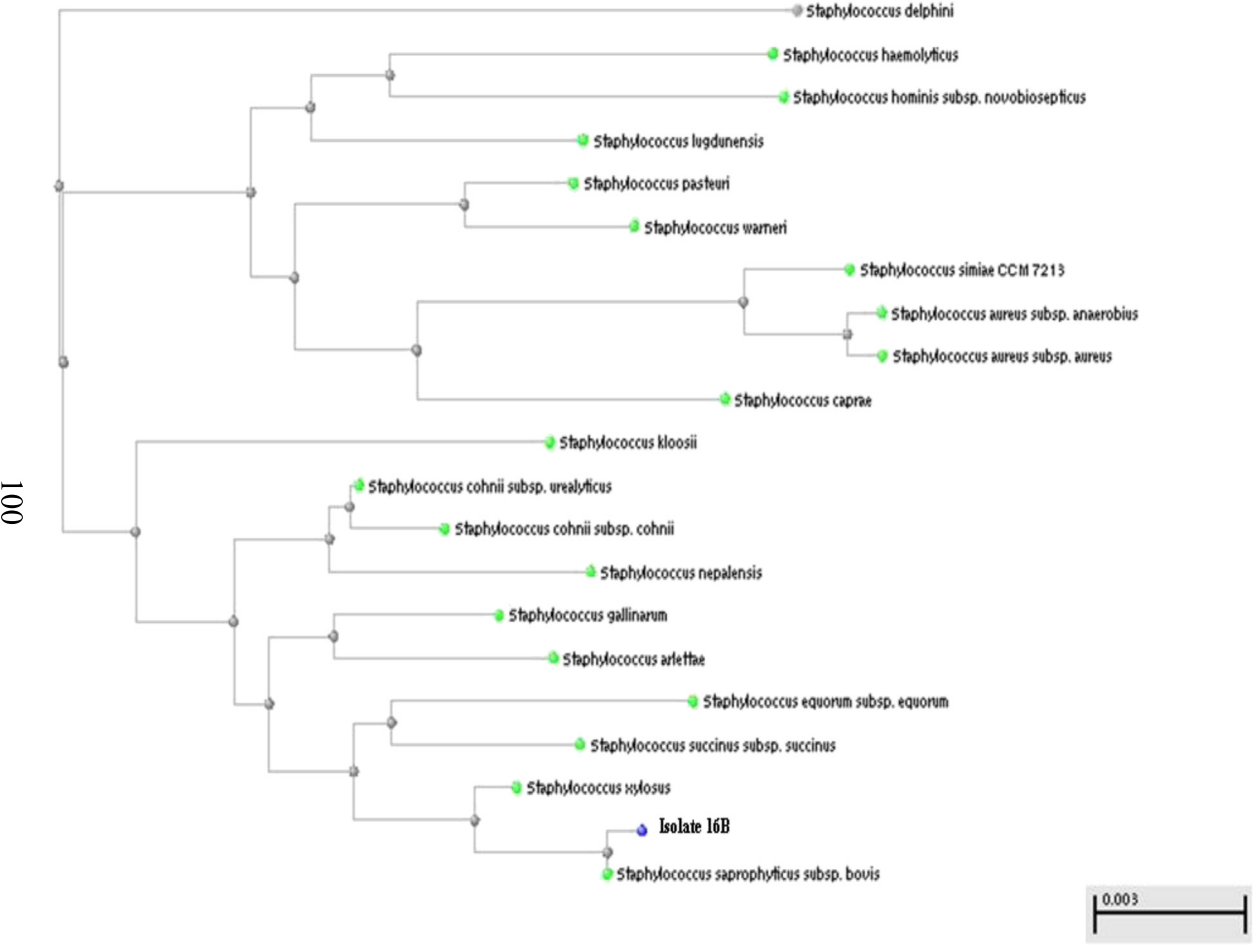
Phylogenetic tree for the Gram-positive isolate 16B

**Fig. 3:**
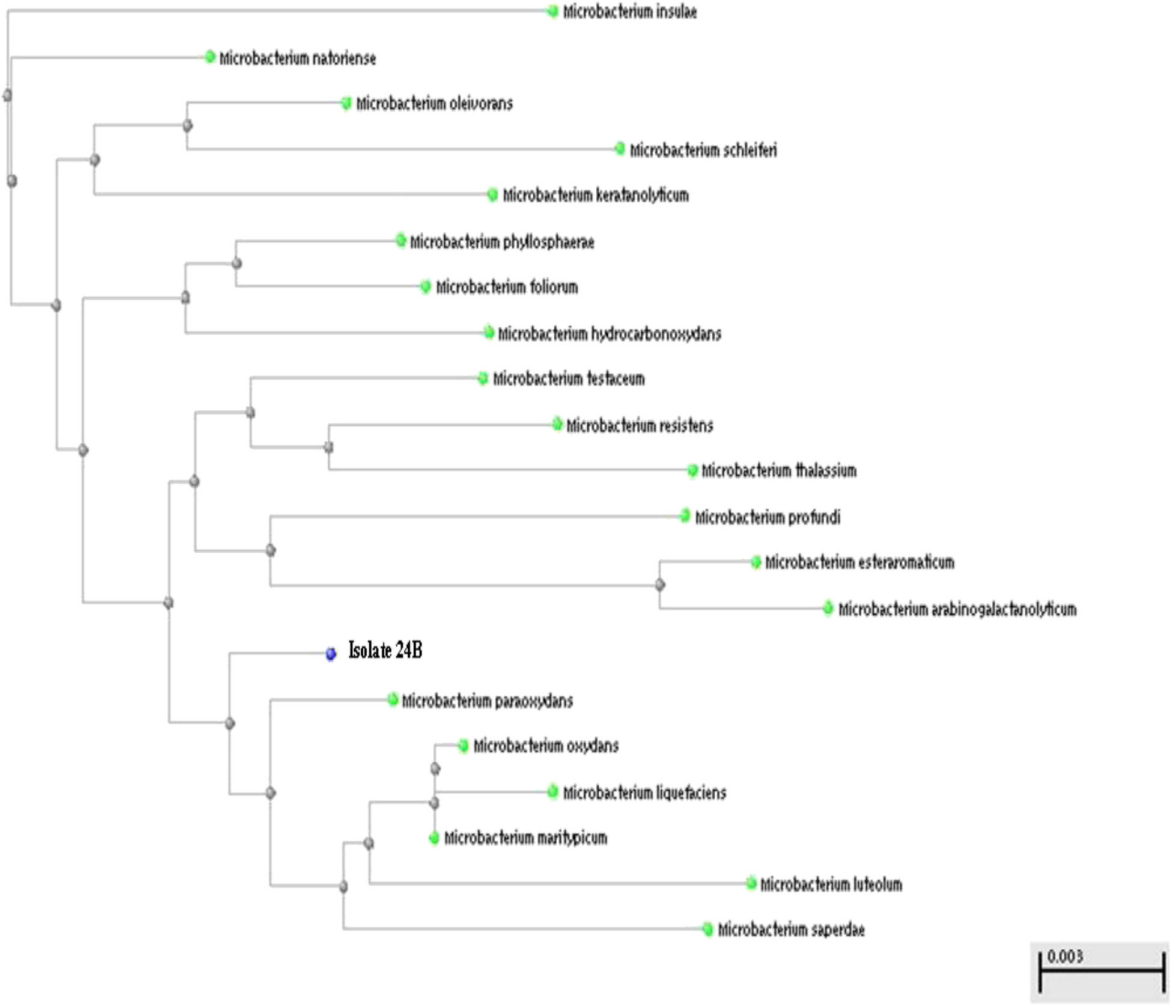
Phylogenetic tree for the Gram-positive isolate 24B

**Fig. 4:**
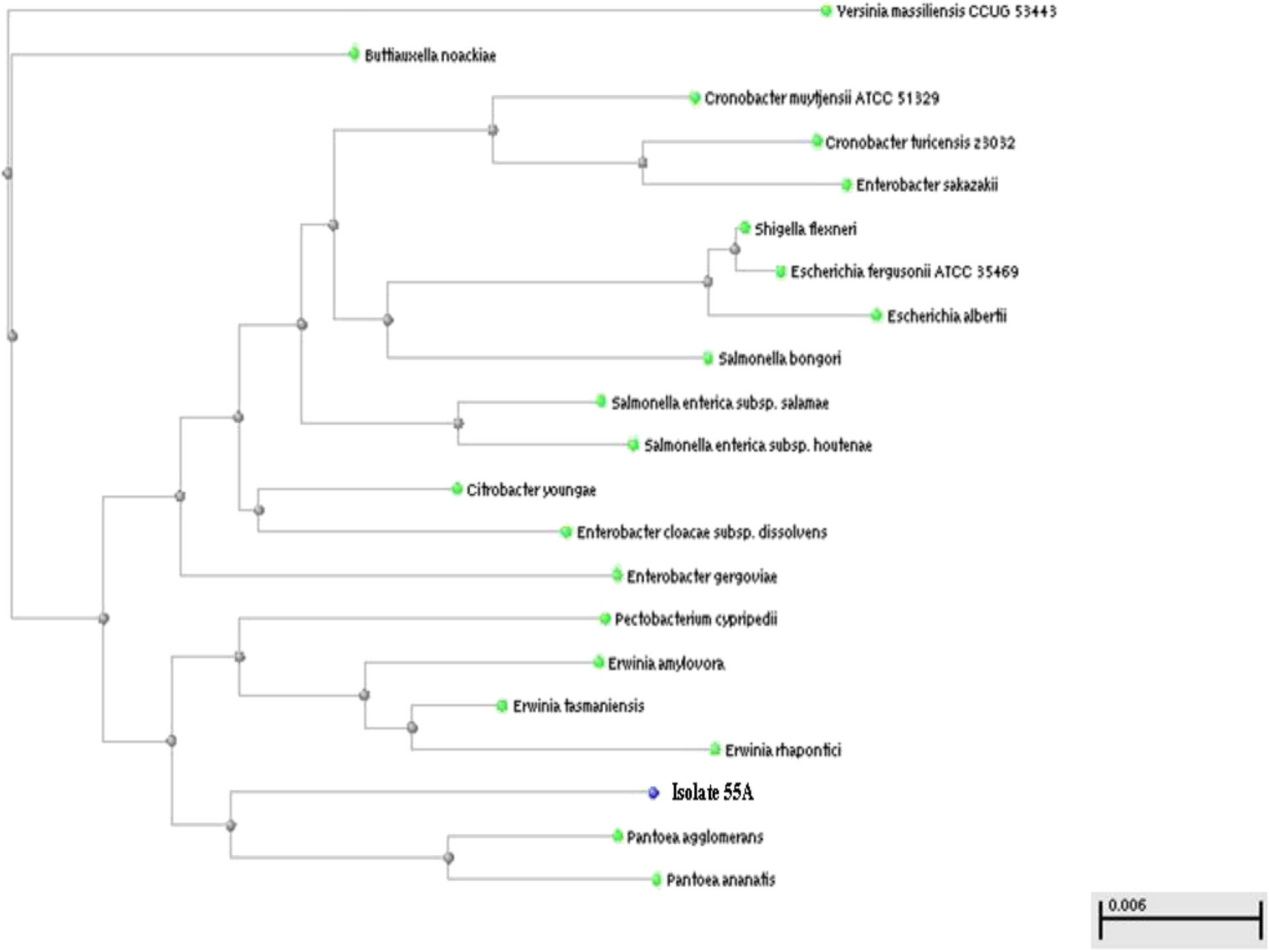
Phylogenetic tree for the Gram-negative isolate 55A

On the other hand, the use of the *16S rRNA* universal primer pair, followed by gene sequencing, was used whenever biochemical identification results were inconclusive. This applied to; (i) two staphylococcal isolates (16B and 22; Table 2), (ii) the Gram-positive non-spore-forming rod-shaped isolate 24B, and (iii) three Gram-negative isolates (11B, 55A and 82A; Table 3). Their identification was achieved by PCR amplification of the conserved *16S rRNA* gene (~1500 bp) using a universal primer pair, followed by DNA sequencing of the amplified *16S rRNA* gene and construction of phylogenetic trees using the BLAST program. Only three figures representing the three groups mentioned above are shown here (Figs. 2, 3 and 4).

The Gram-positive isolate 16B was found to bear similarity to *S. saprophyticus* subsp. *bovis* (phylogenetic tree shown in Fig. 2). This finding coincides with that of the API Staph kit, although the latter’s results were considered “invalid” (Table 2).

Using phylogenetic analysis (Additional file 6), the Gram-positive isolate 22 was found to be closely related to *S. warneri*. Since most of *S. warneri* strains are known to be mannitol-fermentors [38], while this isolate was found to be both mannitol non-fermenting and novobiocin-sensitive, it was incorrectly identified by conventional biochemical methods as *S. epidermidis*. On the other hand, results of the API Staph for the same isolate showed an invalid identification giving a %ID of 46 % and 21.3 % for *S. hominis* and *S. warneri*, respectively (Table 2). Therefore, the molecular identification results of the *S. warneri* isolate 22 somewhat agreed with those of API Staph kit; however, they contradicted those of conventional biochemical tests.

As for the Gram-positive, non-spore-forming, rod-shaped isolate 24B, it was found to be closely related to *Microbacterium paraoxydans* by phylogenetic analysis (Fig. 3). The identity of this isolate, which did not grow on mannitol salt agar nor MacConkey’s agar, could not be determined using the conventional biochemical tests.

Regarding Gram-negative isolates, the phylogenetic tree of isolate 11B (Additional file 7) revealed a close relationship to *Pectobacterium cypripedii*. The genus *Pectobacterium*, which includes Gram-negative non-lactose-fermentors, is closely related to the genus *Erwinia* [39]. The molecular identification result is therefore close to that of conventional biochemical tests, which identified this isolate as *Erwinia cacticida*. On the other hand, the API 20E kit identified the same isolate, though to a low discrimination level, as *Buttiauxella agrestis* (Table 3). This result confirms the fact that the API 20E kit does not consider lactose fermentation in the differentiation between members of the family Enterobacteriaceae.

The phylogenetic relationship between the Gram-negative isolate 55A and *Pantoea agglomerans* is illustrated in Fig. 4. This result was in accordance with that of the API 20E kit that identified this isolate as *Pantoea* spp. (%ID = 50.7), but with a low level of discrimination. While *K. pneumoniae subsp. pneumoniae* (%ID = 30.4) came in second place in the API kit identification, it did not even appear in the phylogenetic tree of the isolate.

Finally, the Gram-negative isolate 82A was found to be closely related to *P. stutzeri* LMG 11199T (Additional file 8), which, in turn, is in accordance with conventional biochemical tests, where it was identified as *Pseudomonas* spp. other than *P. aeruginosa* (Table 3). It is noteworthy that this isolate was not conclusively identified by the API 20E system where it was merely described as a non-fermentor species with %ID = 32.1,and a low discrimination level.

### Artificial contamination of pharmaceutical samples

Biochemical methods for detecting bacterial contaminants in the pharmaceutical samples involved streaking onto selective/differential agar media for isolation of the target microorganisms. Representative well-isolated bacterial colonies were then transferred to TSA plates for further morphological and biochemical confirmatory identification tests. The standard strains *S. aureus* ATCC 6538P and *E. coli* NCTC 10418 were subjected to biochemical analysis using the API identification system. *S. aureus* was misidentified as *S. xylosus* with a good identification level, probably due to the fact that the API Staph kit does not include the coagulase test. On the other hand, *E. coli* was correctly identified using the API 20E kit, with a very good identification level.

In our study, DNA extraction was achieved by boiling, as suggested by Jimenez et al. [40], since that method was found to be effective for all of the tested bacterial contaminants. The specificity of the different previously reported DNA primer pairs used was confirmed using a BLAST search [41], available through the website of the National Centre for Biotechnology Information (NCBI).

The working concentration of the primer pairs specific to *P. aeruginosa*, *E. coli* and *S. aureus* used in PCR was 10 picomoles/μl. However, the *S. enterica*-specific primer pair was used at a higher concentration (20–100 picomoles/μl), in order to show a PCR product.

**Fig. 5:**
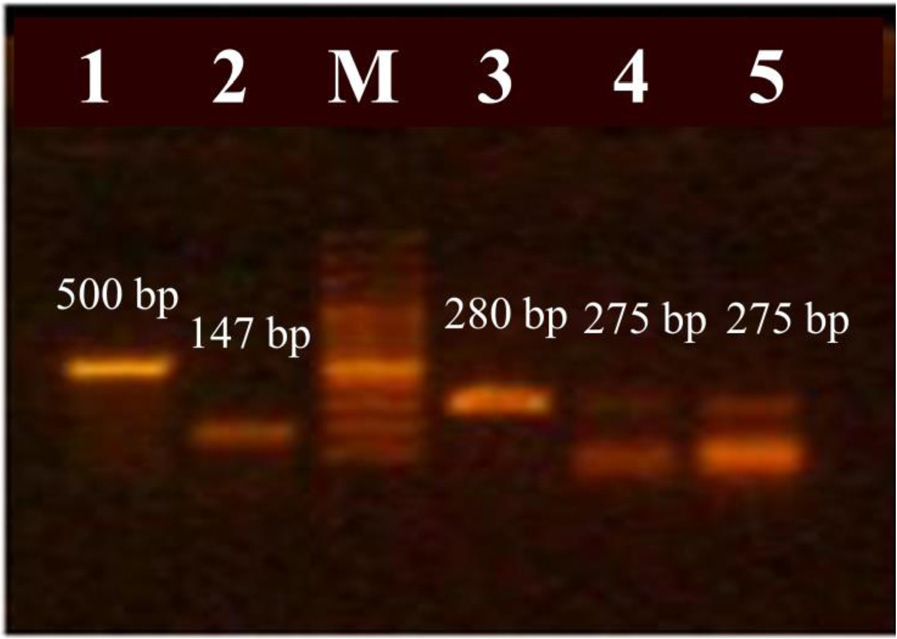
PCR amplification of specific gene fragments in selected strains. PCR amplification products of the *oprL* gene fragment (500bp) in *P. aeruginosa* ATCC 9027 (Lane 1); *uidA* gene fragment (147 bp) in *E. coli* NCTC 10418 (Lane 2); *nucA* gene fragment (280 bp) in *S. aureus* ATCC 6538P (Lane 3) and *invA* gene fragment (275 bp) in *Sal. enterica* ATCC 14028 using 20 picomoles/μL and 100 picomoles/μL species-specific primer pair, respectively. Lane M shows 100 bp plus DNA ladder of fragments sizes (bp; from top to bottom): 3000, 2000, 1500, 1200, **1000**, 900, 800, 700, 600, **500**, 400, 300, 200 and 100

All of the tested bacterial contaminants were successfully detected in their respective glucose samples by PCR amplification of respective characteristic DNA fragments using species-specific primer pairs (Fig. 5). It should be noted that the PCR product of *S. enterica* was more intense when using the primer pair at a higher concentration (100 picomoles/μl); however, a strong primer dimer band was detected.

A period of 5–7 days was required for the bacteria to be isolated, purified, and identified by biochemical methods. On the other hand, the time needed to complete the PCR assay, including both sample preparation and PCR amplification of the specific DNA bacterial targets, was only 29–30 h.

The calculated cost of each method included all the steps required to achieve final identification of the selected bacterial contaminants. However, it should be noted that the cost of equipment required for each method was not included. It was found that the cost of culture media, reagents and kits required for the identification of the selected bacterial contaminants by biochemical methods in 4 samples mount to about 373 Egyptian Pounds (L.E.). On the other hand, the cost of the reagents and chemicals required for the PCR-based assay was about 22.7 L.E. for all 4 samples. Although the individual reagents for the PCR assay are relatively more expensive, they are much less in number.

We then estimated the MDL for each identification method under investigation by inoculating 10 % glucose solutions with different inocula (CFU/ml) of the standard *E. coli* NCTC 10418. On one hand, the inoculated glucose samples were streaked onto selective agar media as well as TSA plates to determine the least inoculum exhibiting growth. On the other hand, the minimum inoculum showing a detectable PCR band of the specified size on agarose gel was determined by pelleting 1 ml of each inoculated glucose sample and using the pellet as a source of DNA (by heating at 95 °C for 10 min), in order to exclude the effect of the DNA extraction step on the determination of the MDL of the PCR-based method.

We found that the MDL of *E. coli* using the PCR assay (10^4^ CFU/ml of sample) was lower than that observed when using conventional methods (10^5^ CFU/ml of sample).

## Discussion

Microbial contamination of pharmaceuticals may arise during manufacturing, storage or use by the consumer, and can ultimately result in several undesirable consequences. In Egypt, microbiological labs follow the USP and BP recommendations for testing the presence/load of microbial contaminants in non-sterile dosage forms and dietary supplements.

In the present study, different biochemical (conventional and API) and molecular (PCR-based) methods were used for the identification of a number of bacterial contaminants isolated from various types of pharmaceuticals (including pharmaceutical preparations, cosmetic preparations, herbal products and raw materials). Both of these methods were then compared with regard to the recovery and identification of selected bacterial contaminants.

The bacterial contaminants were isolated from 31 out of 85 pre-used pharmaceutical products tested (36.5 %). Similarly, it was reported earlier that 50% of the nonsterile pharmaceuticals products tested were heavily contaminated [42]. On the other hand, Campana et al. [1] reported much lower contamination of the tested cosmetic products (10.6 %). Pre-used products were chosen for this study, in order to increase the probability of finding contaminants. Contaminants isolated from pharmaceutical preparations, cosmetics and raw materials were mostly Gram-positive (~90.5 %, 19 out of 21 contaminants), while those isolated from herbal products were mostly Gram-negative (90 %, 9 out of 10 contaminants). In agreement with this latter finding, Abba et al. reported that 46.67 % of herbal remedies tested were contaminated with *S. typhi*, 19.33 % with *Shigella spp.* and 58.67 % with *E. coli* [43]. The observed contamination of herbal tea bags in this study with Gram-negative bacteria could be due to any of the following reasons: (i) unsuitable preparation methods; (ii) contaminated materials and equipment; and/or (iii) improper handling of raw materials by infected personnel during processing. In order for herbal preparations to maintain best quality, safety and efficacy, manufacturing companies must ensure the highest level of hygiene during manufacturing, in order to ensure the lowest possible level of pathogenic organisms in their final herbal products. On the other hand, incidents of microbial contamination of cosmetics are widely reported [44–46]. This could be attributed to inadequate preservation of cosmetics or the use of expired products by the public, which in turn can lead to microbial contamination that favors growth and proliferation of skin pathogens upon use [47]. In 2003, Hugbo and colleagues reported that microbial contamination was detected in some brands of cosmetic creams, where the most common bacterial contaminants found were *Staphylococcus spp.* and *Bacillus spp*., a finding which is similar to ours in the current study [48]. On the other hand, a recent study conducted on non-sterile oral dosage forms found that contaminants were mostly Gram-negative enterobacteria [49].

Compared to cosmetics and pharmaceutical preparations, raw materials showed a higher percentage of contamination (58.8 %), probably due to the lack of effective chemical preservation, where 100 % of the contaminants where Gram-positive bacteria. Our results are in agreement with those of De Clerck et al. who reported that the majority of contaminants isolated from gelatin extracts belonged to members of the genus *Bacillus* [26].

In the current study, 39 % of the contaminants were identified as *Bacillus* spp., the majority of which were isolated from non-sterile dosage forms. Similarly, in a recent study from the year 2010, Mugoyela and Mwambete [42] reported that the majority of microbial contaminants isolated from non-sterile pharmaceuticals were *Bacillus* spp.,

Since only 26.8 % of the isolated contaminants (6 Gram-positive and 5 Gram-negative isolates) were conclusively identified using both conventional methods and the API Staph/API 20E system, molecular identification was deemed necessary for the remaining contaminants. In addition, further identification of *Bacillus* contaminants to their exact species was not feasible by conventional biochemical tests, since members of the genus *Bacillus* exhibit a wide range of physiologic versatility, allowing them to live in almost every natural environment [26, 50]. They therefore required further identification using PCR-based methods using species-specific primer pairs. Similar to conventional biochemical test results, isolates 20A and 52 showed a PCR product at the corresponding band size (174 bp), and were thus identified as *S. epidermidis.* It should be noted that isolate 20A was mistakenly identified as *S. auricularis* using the API Staph kit. Although *S. auricularis* is biochemically similar to *S. epidermidis*, the former is urease-negative [38]. This observed contradiction in the biochemical identification results of isolate 20A most probably arose due to the fact that it gave a positive urease test using the conventional method, whereas it was reported as urease-negative using the API Staph kit. *S. chromogenes* also has a similar biochemical profile to *S. epidermidis*; however, isolate 52 was not identified as *S. chromogenes*, since the latter produces yellow-pigmented colonies upon culturing on nutrient agar [51], unlike isolate 52.

Some identification schemes do not account for phenotypic variations among strains belonging to the same species. For instance, *S. saprophyticus* is listed in several identification schemes as mannitol non-fermenting, although in our study, one mannitol-fermentor, isolate 16B, was conclusively identified as *S. saprophyticus* by phylogenetic analysis. On the other hand, most of *S. warneri* strains are known to be mannitol-fermentors [38]. This fact resulted in the erroneous identification of the *S. warneri* isolate 22, a mannitol non-fermentor, novobiocin-sensitive and urease-positive staphylococcal isolate, as *S. epidermidis* by conventional biochemical methods. Nevertheless, all five *S. epidermidis* isolates (16A, 20A, 20B, 52, 57A) were conclusively identified as such by conventional biochemical tests, based on their mannitol non-fermenting, coagulase- and DNAse-negative, novobiocin-sensitive and urease-positive characteristics.

In this study, both biochemical and PCR-based methods were used to detect bacterial indicators in artificially-contaminated pharmaceutical grade glucose powder, for the purpose of comparing both identification methods with respect to time and cost. Optimization of the PCR reaction conditions for the different primer pairs (annealing temperature: 55°C; extension time: 45 s) was done in the PCR-based identification method, in an attempt to save time. . The PCR reaction conditions optimized in other similar studies [29, 33] differed, probably depending on the primer pairs used and the size of the amplicons produced besides the type of DNA polymerase used for amplification.

The relatively long time required by biochemical methods of identification was similar to other studies [3, 10, 33, 52]. We therefore concluded that the standard USP procedure [53], which relies primarily on biochemical methods for the identification of bacterial indicators, was both time-consuming and labor-intensive. It also requires multiple steps for the growth and isolation of pure bacterial cultures prior to their identification, resulting in delaying the release of raw materials/pharmaceutical products. On the other hand, the relatively short time necessary to conduct a PCR assay was in accordance with previous studies [33, 52]. On estimating the MDL for each identification method, the MDL of *E. coli* using the PCR assay was lower than that observed using conventional methods. Similarly, Jimenez et al. [54] calculated a MDL of 10^4^ CFU/ml for *Salmonella spp*. by a PCR-based method, while a year later, a higher MDL (10^5^ CFU/ml) for *E. coli*, *P. aeruginosa* and *S. aureus* was reported [40]. Conversely, Samadi et al. [55] reported a much lower MDL of 10^2^ CFU/ml. More recently, Farajnia et al. [52] were able to detect microbial contamination at a level of less than 10 CFU/ml or gram of a product, using a multiplex PCR assay. None of the latter studies calculated an MDL for conventional tests.

## Conclusion

PCR-based methods provided an earlier, more cost-effective and more sensitive detection and identification of bacterial contamination, compared to standard biochemical methods currently applied in the pharmaceutical industry. This would allow for rapid implementation of corrective actions, thereby minimizing manufacturing losses and speeding up product release.

## Additional files

Additional file 1:
**Product information of the 85 tested pharmaceuticals.** (DOCX 127 kb) Additional file 2:
**Biochemical tests for the identification of** ***Bacillus*** **isolates. **(DOCX 57 kb) Additional file 3:
**PCR-based identification of staphylococci using species-specific primers.** (DOCX 59 kb) Additional file 4:
**PCR amplification of specific** ***gyr A*** **gene fragment (1027 bp) in** ***B. subtilis*** **.** Lane M: 100 bp plus DNA ladder*; Lane 1: negative control (*B. cereus* ATCC 14579); Lane 2: positive control (*B. subtilis* ATCC 6633); Lane 3: isolate 3A; Lane 4: isolate 3B; Lane 5: isolate 8B; Lane 6: isolate 9; Lane 7: isolate 13; Lane 8: isolate 18; Lane 9: isolate 28; Lane 10: isolate 29; Lane 11: isolate 32; Lane 12: isolate 35; Lane 13: isolate 37; Lane 14: isolate 39; Lane 15: isolate 51; Lane 16: isolate 57B; Lane 17: isolate 63; Lane 18: isolate 82B. * DNA ladder yields 14 fragments. Fragments sizes (bp): 3000, 2000, 1500, 1200, 1000, 900, 800, 700, 600, 500, 400, 300, 200 and 100. (DOCX 763 kb) Additional file 5:
**PCR amplification of specific** ***gyr B*** **gene fragment (364 bp) in** ***B. cereus.*** Lane M: 100 bp plus DNA ladder*; Lane 1: negative control (*B. subtilis* ATCC 6633); Lane 2: positive control (*B. cereus* ATCC 14579); Lane 3: isolate 3A; Lane 4: isolate 3B; Lane 5: isolate 8B; Lane 6: isolate 9; Lane 7: isolate 13; Lane 8: isolate 18; Lane 9: isolate 28; Lane 10: isolate 29; Lane 11: isolate 32; Lane 12: isolate 35; Lane 13: isolate 37; Lane 14: isolate 39; Lane 15: isolate 51; Lane 16: isolate 57B; Lane 17: isolate 63; Lane 18: isolate 82B. * DNA ladder yields 14 fragments. Fragments sizes (bp): 3000, 2000, 1500, 1200, **1000**, 900, 800, 700, 600, **500**, 400, 300, 200 and 100. (DOCX 444 kb) Additional file 6:
**Phylogenetic tree for the Gram-positive isolate 22.** (DOCX 134 kb) Additional file 7:
**Phylogenetic tree for the Gram-negative isolate 11B.** (DOCX 140 kb) Additional file 8:
**Phylogenetic tree for the Gram-negative isolate 82A.** (DOCX 142 kb)

## Abbreviations

DiW: Deionized water
L.E.: Egyptian Pounds
MSA: Mannitol salt agar
PCR: Polymerase chain reaction
PLT: Polysorbate-20 5% v/v, lecithin 0.3% w/v, thioglycolate 0.01% w/v
TSA: Trypticase soy agar
TSB: Tryptic soy broth
USP: United States Pharmacopeia
XLD: Xylose-Lysine-Desoxycholate
μl: microliters
