# Supplementary material for: Microbiological testing of pharmaceuticals and cosmetics in Egypt
Source: BMC Microbiol. 2015 Dec 9;15:275. doi: 10.1186/s12866-015-0609-z (PMC4674922; doi:10.1186/s12866-015-0609-z)
Supplement: Additional file 1: — Product information of the 85 tested pharmaceuticals. (DOCX 127 kb) [file 12866_2015_609_MOESM1_ESM.docx]

**Product information of the 85 tested pharmaceuticals**

| **Product code** | **Product name** | **Constituents** | **Description and dosage form** | **Manufacturing company (Country)** | **Batch number** | **Expiry date** | **Date of sample collection** |
| --- | --- | --- | --- | --- | --- | --- | --- |
| **1** | **Sekem flu** | tilia, guava, verbascum, majoram, peppermint, fennel, liquorice | teabags;  1.5 gm/bag | Sekem (Egypt) | HS0209/08 | 12/10 | 26/05/09 |
| **2** | **Afrin nasal spray** | oxymetazoline HCl | nasal spray;  20 mL | MUP (Egypt) | 672202 | 10/10 | 26/05/09 |
| **3** | **Bronchicum^®^ elixir S** | grindelia, pimpinella, primula, thymus, quebracho | herbal preparation for cough, food supplement, elixir; 100 mL | Sanofi Aventis s.a.e (Egypt) | 18502 | 01/10 | 26/05/09 |
| **4** | **Calamyl_D_** | calamine, diphenhydramine HCl, zinc oxide, camphor | lotion; 60 mL | MUP (Egypt) | 060889 | 04/09 | 2/06/09 |
| **5** | **Berberil^®^ N ophtiole** | tetrahydrozoline HCl | sterile eye drops; 10 mL | Mina Pharm (Egypt) | 8CE0323 | 02/11 | 2/06/09 |
| **6** | **Prisoline drops** | naphazoline HCl, chlorpheniramine maleate | eye and nasal drops, isotonic buffer solution; 15 mL | Kahira Pharmaceuticals & Chemical Industries Company (Egypt) | 0761293 | 11/10 | 6/06/09 |
| **7** | **Glysolid cream** | water, glycerin, cetearyl alcohol, allantoin, decyl oleate, ceteareth-12, ceteareth-20, sodium cetearyl sulfate, dimethicone, silica, fragrance, butylene glycol, iodopropynyl butyl carbamate, DMDM hydantoin | glycerin hand cream; 40 mL | Pyramids Cosmetics Company (Egypt) | NA | NA | 2/06/09 |

**Product information of the 85 tested pharmaceuticals (cont.)**

| **Product code** | **Product name** | **Constituents** | **Description and dosage form** | **Manufacturing company (Country)** | **Batch number** | **Expiry date** | **Date of sample collection** |
| --- | --- | --- | --- | --- | --- | --- | --- |
| **8** | **Perfect^®^ care** | polyhexanide 0.001 mg/mL, NaCl, poloxamer 407, disodium hydrogen phosphate, sodium dihydrogen phosphate, disodium edetate | sterile isotonic contact lens solution for soft lens;  60 mL | Kahira Pharmaceuticals & Chemical Industries Company (Egypt) | 86323 | 03/10 | 2/06/09 |
| **9** | **Toplexil** | oxomemazine, glyceryl guaiacolate, sodium benzoate, paracetamol | cough relieving and expectorant syrup; 125 mL | European Egyptian Pharmaceutical Industries (Egypt) | 7523204 | 11/12 | 7/06/09 |
| **10** | **D-galactose** | D-galactose | powder | NA | NA | NA | 4/07/09 |
| **11** | **Sekem diarrhea** | achillea, chamomile, basil, vine leaves, verbascum, hibiscus, chicory | teabags | Sekem (Egypt) | HS0031/08 | 02/11 | 4/07/09 |
| **12** | **Sekem rheume** | salix cortex, urtica, taraxacum, parsley, peppermint, ambrosia, chamomile | teabags | Sekem (Egypt) | HS0161/07 | 10/10 | 4/07/09 |
| **13** | **Kokuryu** | NA | foundation;  50 gm | Cherry Lab. Inc. (Philippines) | NA | NA | 7/07/09 |
| **14** | **Laprairie cleansing emulsion** | NA | emulsion;  10 mL | Laprairie Inc. (Switzerland) | 9300280 | NA | 7/07/09 |
| **15** | **Color trend crystal clear mascara** | NA | transparent mascara; 7 mL | Avon (Egypt) | Z201107 | NA | 7/07/09 |
| **16** | **Lipgloss** | NA | viscous liquid with a brush | Miss Rose (Egypt) | NA | NA | 7/07/09 |

**Product information of the 85 tested pharmaceuticals (cont.)**

| **Product code** | **Product name** | **Constituents** | **Description and dosage form** | **Manufacturing company (Country)** | **Batch number** | **Expiry date** | **Date of sample collection** |
| --- | --- | --- | --- | --- | --- | --- | --- |
| **17** | **Tears guard eye drops** | hydroxypropyl-methylcellulose  3 mg/mL | sterile eye drops | Kahira Pharmaceuticals & Chemical Industries Company (Egypt) | 86350 | 03/10 | 22/07/09 |
| **18** | **Bron king** | mixture of standardized natural extracts of thyme leaves, guava leaves, tilia flowers and fennel oil with purified honey | cough syrup; 120 mL | Sigma Pharmaceutical Industries (Egypt) | 71569 | 10/10 | 27/07/09 |
| **19** | **Viscéralgine spasmolytic** | tiemonium | syrup; 120 mL | SEDICO Pharmaceutical Co. (Egypt) | 1107629 | 11/10 | 27/07/09 |
| **20** | **Bricanyl** | terbutaline sulfate 30 mg,  Spir. Fort 200 mg, sorbitol 15 gm, aroma, purified water | syrup; 120 mL | Chemical industries development, CID (Egypt) | 115 | 02/10 | 27/07/09 |
| **21** | **Mucogel** | dried aluminium hydroxide gel, magnesium hydroxide, oxethazine | suspension; 180 mL | E.I.P.I.CO (Egypt) | 056370 | 08/09 | 27/07/09 |
| **22** | **Sucrose powder** | sucrose | white crystals | The Arab Company for Gelatin and Pharmaceutical Products (Egypt) | 2009/14 | NA | 1/08/09 |
| **23** | **Ginger powder** | ginger | powder |  | 10080609 | NA | 1/08/09 |
| **24** | **Gelatin powder** | gelatin | beige crystals |  | 6234 | NA | 1/08/09 |
| **25** | **Atropine** | atropine | powder;  100 gm | NA | NA | NA | 1/08/09 |

**Product information of the 85 tested pharmaceuticals (cont.)**

| **Product code** | **Product name** | **Constituents** | **Description and dosage form** | **Manufacturing company (Country)** | **Batch number** | **Expiry date** | **Date of sample collection** |
| --- | --- | --- | --- | --- | --- | --- | --- |
| **26** | **Hyoscyamine extract** | hyoscyamine | powder | NA | NA | NA | 1/08/09 |
| **27** | **Ephedrine alkaloid USP** | ephedrine | crystals; 25 gm | Merck & Co., Inc. (USA) | 40567 | NA | 1/08/09 |
| **28** | **Starch** | starch, ash not more than 0.5% | White, water-soluble powder | Nasr Company for Pharmaceutical Chemicals (Egypt) | NA | NA | 25/08/09 |
| **29** | **Sekem cough** | thyme, fennel, liquorice, salvia, eucalyptus, verbascum, melisse | teabags;  1.5 gm/ bag | Sekem (Egypt) | HS0048/09 | 02/12 | 21/04/10 |
| **30** | **Bringo lotion** | zinc oxide, extract of thyme, extract of chamomile, menthol, camphor, glycerin | lotion; 120 mL | Macro International Group (Egypt) | 0661 | 06/08 | 21/04/10 |
| **31** | **Enteroquin compound** | Diiodohydroxy quinoline, phthalyl sulphathiazole, sulphadimidine, kaolin light, vitamin B_1_, vitamin B_2_, vitamin B_6_, calcium pantothenate, nicotinamide, homatropine methyl bromide | powder for oral suspension | ADCO (Egypt) | 580047 | 09/09 | 21/04/10 |
| **32** | **Gelatin powder BP 63** | gelatin | gelatin powder | NA | NA | NA | 27/04/10 |
| **33** | **Aspirin BP80** | aspirin | white crystals | NA | NA | NA | 27/04/10 |
| **34** | **Citric acid** | citric acid | colorless crystals | NA | NA | NA | 27/04/10 |

**Product information of the 85 tested pharmaceuticals (cont.)**

| **Product code** | **Product name** | **Constituents** | **Description and dosage form** | **Manufacturing company (Country)** | **Batch number** | **Expiry date** | **Date of sample collection** |
| --- | --- | --- | --- | --- | --- | --- | --- |
| **35** | **D(+)Glucose anhydrous** | anhydrous glucose | white powder; 1 kg | Prolabo Products for Lab Rhone-poulenc (France) | 7533026 | NA | 27/04/10 |
| **36** | **Kieselguhr** | kieselguhr, iron 0.05%, heavy metals (as Pb) 0.005%, chloride 0.01%, sulphate 0.01% | white fluffy powder; 1kg | Riedel-de Haën AG (Germany) | 465952 | NA | 27/04/10 |
| **37** | **Calamine (raw material)** | calamine | pink powder; 100 gm | NA | NA | NA | 16/05/10 |
| **38** | **Lactose** | lactose | powder | NA | NA | NA | 16/05/10 |
| **39** | **Sucrose** | sucrose | white crystals; 100 gm | Difco Laboratories Incorporated (USA) | NA | NA | 16/05/10 |
| **40** | **Glycine** | glycine | powder | NA | NA | NA | 16/05/10 |
| **41** | **Gripe water** | terpeneless dill seed oil, sodium bicarbonate | syrup; 120 mL | Sigma Pharmaceutical Industries (Egypt) | 82117 | 10/11 | 16/05/10 |
| **42** | **Paramol** | paracetamol | syrup; 125 mL | Misr Company For Pharmaceutical Industries (Egypt) | 423069 | 06/12 | 15/06/10 |
| **43** | **Bisolvon elixir** | bromhexine hydrochloride | elixir; 115 mL | CID (Egypt) | 189 | 12/11 | 15/06/10 |
| **44** | **Visions freaking amazing mascara** | NA | black mascara; 8 mL | Oriflame (Poland) | FE 9W1 | 06/12 | 15/06/10 |

**Product information of the 85 tested pharmaceuticals (cont.)**

| **Product code** | **Product name** | **Constituents** | **Description and dosage form** | **Manufacturing company (Country)** | **Batch number** | **Expiry date** | **Date of sample collection** |
| --- | --- | --- | --- | --- | --- | --- | --- |
| **45** | **Oriflame power curl mascara** | NA | black mascara; 8 mL | Oriflame (Poland) | MAD8W1 | 11/11 | 15/06/10 |
| **46** | **Oriflame wonder lash mascara** | NA | black mascara; 8 mL | Oriflame (Poland) | NA | NA | 15/06/10 |
| **47** | **Giordani pearls** | NA | bronzing pearls; 25 gm | Oriflame (Sweden) | NA | 06/01 | 15/06/10 |
| **48** | **True match™ super-blendable powder** | talc, corn starch, dimethicone, zinc stearate, pentaerythrityl tetraisostearate, octyldodecylstearoyl stearate, zeolite, sorbic acid, methyl paraben, propyl paraben, tocopheryl acetate, tetrasodium EDTA, butyl paraben, BHT, panthenol | light ivory face powder;  9.5 gm | L’Oreal Paris (USA) | WD036 | NA | 15/06/10 |
| **49** | **Oriflame complete perfection 2 in 1 foundation** | NA | foundation;  7 gm | Oriflame (Germany) | MB0 | 03/11 | 15/06/10 |
| **50** | **Ҫiao lipgloss** | NA | lipgloss | Ҫiao (Egypt) | 509 | 05/09 | 21/06/10 |
| **51** | **Oriflame flawless pressed powder** | NA | compact powder; 10 gm | Oriflame (Italy) | DA5S1 | 01/08 | 21/06/10 |
| **52** | **Oriflame sunglow powder** | NA | compact powder; 4 gm | Oriflame (Germany) | AA6K1B | 01/09 | 21/06/10 |

**Product information of the 85 tested pharmaceuticals (cont.)**

| **Product code** | **Product name** | **Constituents** | **Description and dosage form** | **Manufacturing company (Country)** | **Batch number** | **Expiry date** | **Date of sample collection** |
| --- | --- | --- | --- | --- | --- | --- | --- |
| **53** | **Visions sheer pressed powder** | NA | pressed powder; 9 gm | Oriflame (England) | HA4S1 | 07/07 | 21/06/10 |
| **54** | **Royal regime tea** | 20% *Cichorium intybus* herb, 30% *Cassia angustifolia* leaves, 50% *Foeniculum vulgare* fruits | teabags | Ottoman (Royal for Herbs) (Egypt) | NA | NA | 21/06/10 |
| **55** | **Sekem laxative** | senna, liquorice, chamomile, fennel, dill, coriander, anise | teabags;  2 gm/bag | Sekem (Egypt) | HS0099/09 | 06/11 | 21/06/10 |
| **56** | **Sekem flu** | tilia, guava, verbascum, majoram, peppermint, fennel, liquorice | teabags;  1.5 gm/bag | Sekem (Egypt) | HS0064/09 | 03/11 | 21/06/10 |
| **57** | **Feather finish cream powder refill** | NA | honey beige compact powder; 20 gm | Yardley (England) | 5/2000 | 08/03 | 21/06/10 |
| **58** | **Nivea volume shine lipgloss** | NA | lipgloss | Nivea (Germany) | 54430054 | NA | 20/07/10 |
| **59** | **Dior addict ultragloss** | NA | lipgloss; 6 mL | Christian Dior Paris (France) | NA | NA | 20/07/10 |
| **60** | **JP mascara** | NA | black mascara | NA | NA | NA | 20/07/10 |
| **61** | **Clarins eye liner** | NA | black eyeliner | Clarins (USA) | NA | NA | 20/07/10 |
| **62** | **Eye shadow Duo Capability brown** | NA | powder | Yardley (England) | 5075 9187 | NA | 20/07/10 |

**Product information of the 85 tested pharmaceuticals (cont.)**

| **Product code** | **Product name** | **Constituents** | **Description and dosage form** | **Manufacturing company (Country)** | **Batch number** | **Expiry date** | **Date of sample collection** |
| --- | --- | --- | --- | --- | --- | --- | --- |
| **63** | **Cybele eye shadow** | NA | silver/blue powder; 5 gm | Cybele Cosmetics Ltd. (Italy) | NA | NA | 20/07/10 |
| **64** | **Motinorm** | domperidone | suspension;  125 mL | GlaxoSmithKline S.A.E (Egypt) | 072138A | 06/09 | 21/07/10 |
| **65** | **Paxeladine** | oxeladine citrate | syrup; 125 mL | ADCO (Egypt) | 530277 | 07/08 | 21/07/10 |
| **66** | **Simethicone drops USP 29** | simethicone | oral drops for infants; 30 mL | Amriya Pharm. Industries (Egypt) | 256702 | 06/10 | 21/07/10 |
| **67** | **Locacorten^®^ vioform ear drops** | flumetasone, pivalic acid, clioquinol | ear drops;  7.5 mL | Novartis Pharma S.A.E (Egypt) | 056 | 07/08 | 21/07/10 |
| **68** | **Amanda blusher** | NA | blusher | Amanda (Egypt) | 21/02 | 02/08 | 21/07/10 |
| **69** | **Magnolia blossom body cream** | water, mineral oil, isopropyl palmitate, stearic acid, glyceryl stearate SE, glyceryl stearate, fragrance, cetyl alcohol, aloe vera leaf juice, hops extract, rosemary leaf extract, lemon fruit extract, pine cone extract, papain, bromelain, calendula officinalis flower oil, avocado oil, sweet almond oil, honey, dimethicone, triethanolamine, propylene glycol, acetylated lanolin, disodium EDTA, methyl and propyl parabens, imidazolidinyl urea, yellow 5, yellow 6, red 4. | cream; 226 gm | Bath & Body works^®^ (USA) | 0276B4B1 | NA | 21/07/10 |

**Product information of the 85 tested pharmaceuticals (cont.)**

| **Product code** | **Product name** | **Constituents** | **Description and dosage form** | **Manufacturing company (Country)** | **Batch number** | **Expiry date** | **Date of sample collection** |
| --- | --- | --- | --- | --- | --- | --- | --- |
| **70** | **Fragrance shimmer body lotion** | water, octyldodecanol, glycerin, mineral oil, glyceryl stearate SE, alcohol, petrolatum, fragrance, titanium dioxide, cyclomethicone, Mica, dimethicone, stearic acid, dicaprylyl ether, myristyl alcohol, hydrolyzed milk, oat proteins, tocopheryl acetate, aloe vera, propylene glycol, camellia, oleifera leaf extract, chamomilla recutita extract, carbomer, sodium hydroxide, phenoxy ethanol, methyl and propyl parabens and iron oxides. | body lotion;  59 mL | Parfums de Coeur^®^ (Canada) | R1241 | 01/09 | 21/07/10 |
| **71** | **Milk and honey face mask** | water, cetearyl alcohol, glycerin, prunus dulcis, liquid paraffin, caprylic/ capric triglyceride, glyceryl stearate, PEG-100 stearate, dimethicone, propylene glycol, xanthan gum, sodium acrylate/ acryloyl dimethyl taurate copolymer, methyl paraben, ceteareth-20, imidazolidinyl urea, isohexadecane, lactic acid, saccharide isomerate, propyl paraben, hydrolyzed milk protein, fragrance, polysorbate 80, sodium hydroxide, Mel, glycolic acid, linalool, sorbitol, sorbitan oleate, phenoxyethanol, hexyl cinnamal, butyl phenyl methyl propional, sodium lactate, benzyl benzoate, citric acid, alpha-isomethyl ionone, citronellol, Ci 47005, Ci 14700 | cream;  50 mL | Oriflame (Poland) | MA5W1 | 11/08 | 21/07/10 |

**Product information of the 85 tested pharmaceuticals (cont.)**

| **Product code** | **Product name** | **Constituents** | **Description and dosage form** | **Manufacturing company (Country)** | **Batch number** | **Expiry date** | **Date of sample collection** |
| --- | --- | --- | --- | --- | --- | --- | --- |
| **72** | **Glycerin hand cream with silicon** | demineralized water, stearic acid, propylene glycol, ethyl hexyl methoxy cinnamate, glycerin, methyl glueth-20 sesquistearate, POE (26m) glycerol, stearyl alcohol, cyclomethicone, methyl glucose sesquistearate, chamomile glycolic extract, marigold extract-glycolic, triethanalamine, bees wax, ginseng extract-glycolic, phenoxyethanol, tocopheryl acetate, methyl *p*-hydroxybenzoate, carbopol, vit.A palmitate, silicon fluid, fragrance, butylated hydroxytoluene | cream; 100 mL | Avon (Egypt) | E2466 | 09/09 | 21/07/10 |
| **73** | **Soothing foot cream** | water, cetearyl alcohol, urea, glycerin, mineral oil, sodium cetearyl sulfate, ceteareth-12, dimethicone, imidazolidinyl urea, fragrance, methyl and propyl parabens, propylene glycol, limonene, hexyl cinnamal, sorbitol, primula vulgaris extract, matricaria flower extract, linalool | cream; 75 mL | Oriflame (India) | DHHH6D | 03/09 | 21/07/10 |
| **74** | **Vidrop** | cholecalciferol (vitamin D_3_) | oral drops;  15 mL | MUP (Egypt) | 104285 | 12/12 | 14/06/11 |
| **75** | **Berberil^®^ N ophtiole** | tetrahydrozoline HCl | sterile eye drops; 10 mL | Mina Pharm (Egypt) | 70E1188 | 07/10 | 14/06/11 |

**Product information of the 85 tested pharmaceuticals (cont.)**

| **Product code** | **Product name** | **Constituents** | **Description and dosage form** | **Manufacturing company (Country)** | **Batch number** | **Expiry date** | **Date of sample collection** |
| --- | --- | --- | --- | --- | --- | --- | --- |
| **76** | **Otrivin** | xylometazoline HCl | nasal drops;  10 mL | Novartis Consumer Health SA (Switzerland) | J00077H | 12/11 | 14/06/11 |
| **77** | **Johnson’s baby oil** | liquid paraffin, isopropyl palmitate, aloe vera, tocopheryl acetate, fragrance | oil; 75 mL | Johnson & Johnson^©^ (Egypt) | 9110 | 10/11 | 14/06/11 |
| **78** | **Phenadone** | dexamethasone, chlorpheniramine maleate | syrup; 125 mL | ADCO (Egypt) | 830141 | 04/11 | 14/06/11 |
| **79** | **Tobrex** | tobramycin | sterile ophthalmic solution; 5 mL | Alcon (Belgium) | 240610 | 05/13 | 14/06/11 |
| **80** | **Amanda velva compact powder** | NA | face compact powder | Amanda (Egypt) | 21 | 05/11 | 14/06/11 |
| **81** | **ADS magic eye shadow** | NA | eye shadow | NA | A3733 | NA | 14/06/11 |
| **82** | **Bourjois blusher visage** | NA | blusher powder;  2.5 gm | Bourjois (France) | NA | NA | 14/06/11 |
| **83** | **Bourjois Pastel Joues blusher** | NA | blusher; 5 gm | Bourjois (France) | 2310 | NA | 14/06/11 |
| **84** | **Tylenol cold multi symptom syrup** | acetaminophen, dextromethorphan hydrobromide, guaifenesin, phenyl ephrine hydrochloride | syrup; 240 mL | Janssen Cilag (Australia) | 905534 | 08/11 | 14/06/11 |
| **85** | **Fenistil** | dimethindene maleate | syrup; 100 mL | Novartis Pharma S.A.E (Egypt) | Y0100 | 02/11 | 14/06/11 |

NA: Not Available.
