# Supplementary material for: Microbiological testing of pharmaceuticals and cosmetics in Egypt
Source: BMC Microbiol. 2015 Dec 9;15:275. doi: 10.1186/s12866-015-0609-z (PMC4674922; doi:10.1186/s12866-015-0609-z)
Supplement: Additional file 2: — Biochemical tests for the identification of Bacillus isolates. (DOCX 57 kb) [file 12866_2015_609_MOESM2_ESM.docx]

**Additional file 2.** Biochemical tests for the identification of *Bacillus* isolates

| **Isolate code(s)** | **Starch hydrolysis test** | **Voges Proskauer test*** | **Catalase test*** | **Conclusion** | |
| --- | --- | --- | --- | --- | --- |
| **3A, 3B, 9, 13, 28, 32, 35, 37, 51, 57B, 63, 82B** | + | + | ND | *B. subtilis*  *B. cereus*  *B. polymyxa*  *B. mycoides*  *B. thuringiensis* | *B. licheniformis*  *B. alvei*  *B. anthracis*  *B. coagulans* |
| **8B, 18, 39** | - | ND | + | *B. badius*  *B. insolitus*  *B. laterosporus* | *B. pasteurii*  *B. marinus*  *B. sphaericus* |
| **29** | + | - | ND | *B. megaterium*  *B. stearothermophilus*  *B. macerans*  *B. pantothenticus*  *B. macquariensis* | *B. lentus*  *B. alcalophilus*  *B. badius*  *B. brevis*  *B. circulans* |

* ND: Not determined
