# Supplementary material for: Microbiological testing of pharmaceuticals and cosmetics in Egypt
Source: BMC Microbiol. 2015 Dec 9;15:275. doi: 10.1186/s12866-015-0609-z (PMC4674922; doi:10.1186/s12866-015-0609-z)
Supplement: Additional file 3: — PCR-based identification of staphylococci using species-specific primers. (DOCX 59 kb) [file 12866_2015_609_MOESM3_ESM.docx]

**Additional File 3.** PCR-based identification of staphylococci using species-specific primers.

| **Isolate code*** | **Species-specific primers^#^** | | | **Conclusion** |
| --- | --- | --- | --- | --- |
|  | ***S. warneri*** | ***S. hominis*** | ***S. epidermidis*** |  |
| **17** | + | - | NA | *S. warneri* |
| **20A** | NA | NA | + | *S. epidermidis* |
| **44** | + | - | NA | *S. warneri* |
| **52** | NA | NA | + | *S. epidermidis* |
| **62** | + | - | NA | *S. warneri* |

* These staphylococci required further identification or showed contradictory results in both conventional biochemical methods and the API Staph kit.

^#^ (+), PCR product at the specified band size; (-), no PCR product at the specified band size;

NA, Not applicable.
