# Supplementary material for: Microbiological testing of pharmaceuticals and cosmetics in Egypt
Source: BMC Microbiol. 2015 Dec 9;15:275. doi: 10.1186/s12866-015-0609-z (PMC4674922; doi:10.1186/s12866-015-0609-z)
Supplement: Additional file 5: — PCR amplification of specific gyr B gene fragment (364 bp) in B. cereus. Lane M: 100 bp plus DNA ladder*; Lane 1: negative control (B. subtilis ATCC 6633); Lane 2: positive control (B. cereus ATCC 14579); Lane 3: isolate 3A; Lane 4: isolate 3B; Lane 5: isolate 8B; Lane 6: isolate 9; Lane 7: isolate 13; Lane 8: isolate 18; Lane 9: isolate 28; Lane 10: isolate 29; Lane 11: isolate 32; Lane 12: isolate 35; Lane 13: isolate 37; Lane 14: isolate 39; Lane 15: isolate 51; Lane 16: isolate 57B; Lane 17: isolate 63; Lane 18: isolate 82B. * DNA ladder yields 14 fragments. Fragments sizes (bp): 3000, 2000, 1500, 1200, 1000, 900, 800, 700, 600, 500, 400, 300, 200 and 100. (DOCX 444 kb) [file 12866_2015_609_MOESM5_ESM.docx]

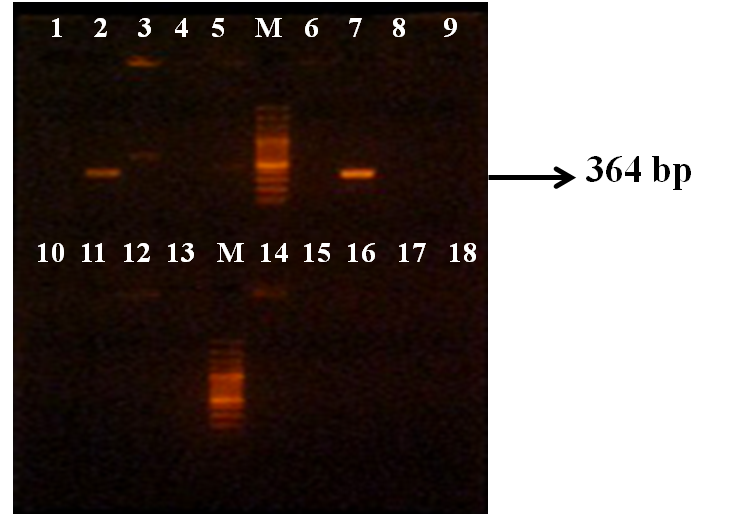


1000 bp

500 bp

1000 bp

500 bp

**Additional file 5:** PCR amplification of specific *gyr B* gene fragment (364 bp) in *B. cereus*

| Lane M: 100 bp plus DNA ladder*  Lane 1: negative control (*B. subtilis* ATCC 6633)  Lane 2: positive control (*B.* *cereus* ATCC 14579)  Lane 3: isolate 3A  Lane 4: isolate 3B  Lane 5: isolate 8B  Lane 6: isolate 9  Lane 7: isolate 13  Lane 8: isolate 18  Lane 9: isolate 28 | Lane 10: isolate 29  Lane 11: isolate 32  Lane 12: isolate 35  Lane 13: isolate 37  Lane 14: isolate 39  Lane 15: isolate 51  Lane 16: isolate 57B  Lane 17: isolate 63  Lane 18: isolate 82B |
| --- | --- |

* DNA ladder yields 14 fragments

Fragments sizes (bp): 3000, 2000, 1500, 1200, **1000**, 900, 800, 700, 600, **500**, 400, 300, 200 and 100.
