# Supplementary figures and images for: Microbiological testing of pharmaceuticals and cosmetics in Egypt
Source: BMC Microbiol. 2015 Dec 9;15:275. doi: 10.1186/s12866-015-0609-z (PMC4674922; doi:10.1186/s12866-015-0609-z)

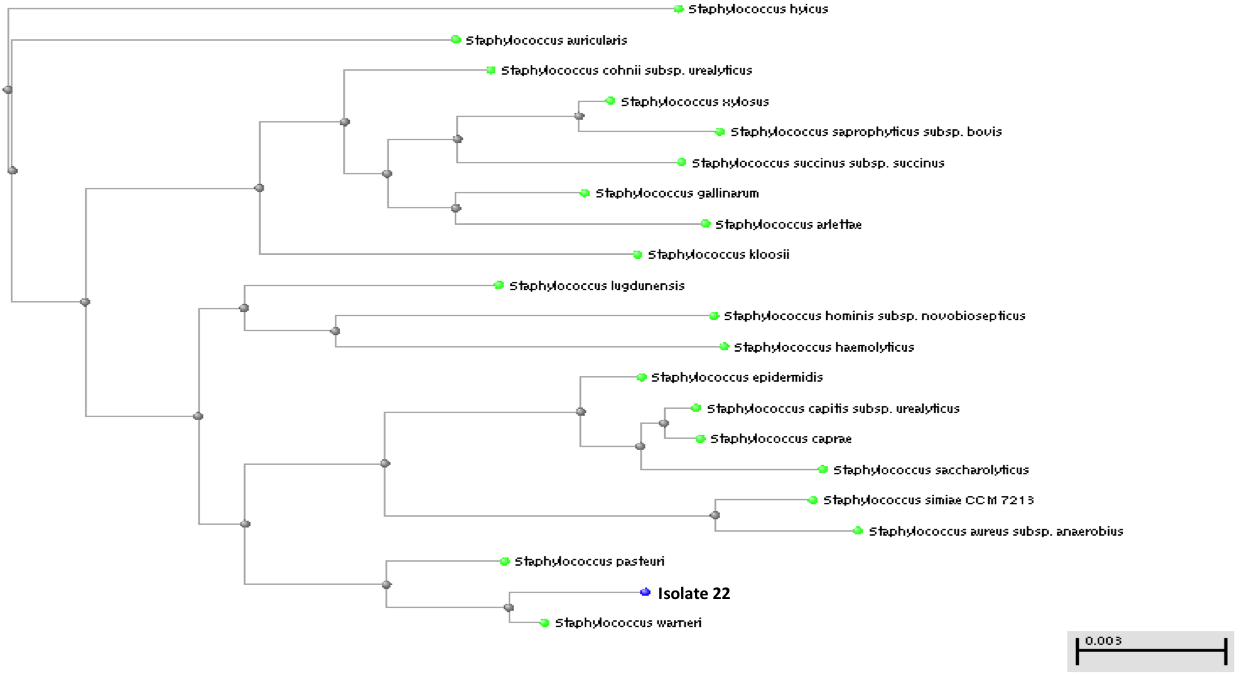


Additional file 6: Phylogenetic tree for the Gram-positive isolate 22

Supplement: Additional file 6: — Phylogenetic tree for the Gram-positive isolate 22. (DOCX 134 kb) [file 12866_2015_609_MOESM6_ESM.docx]

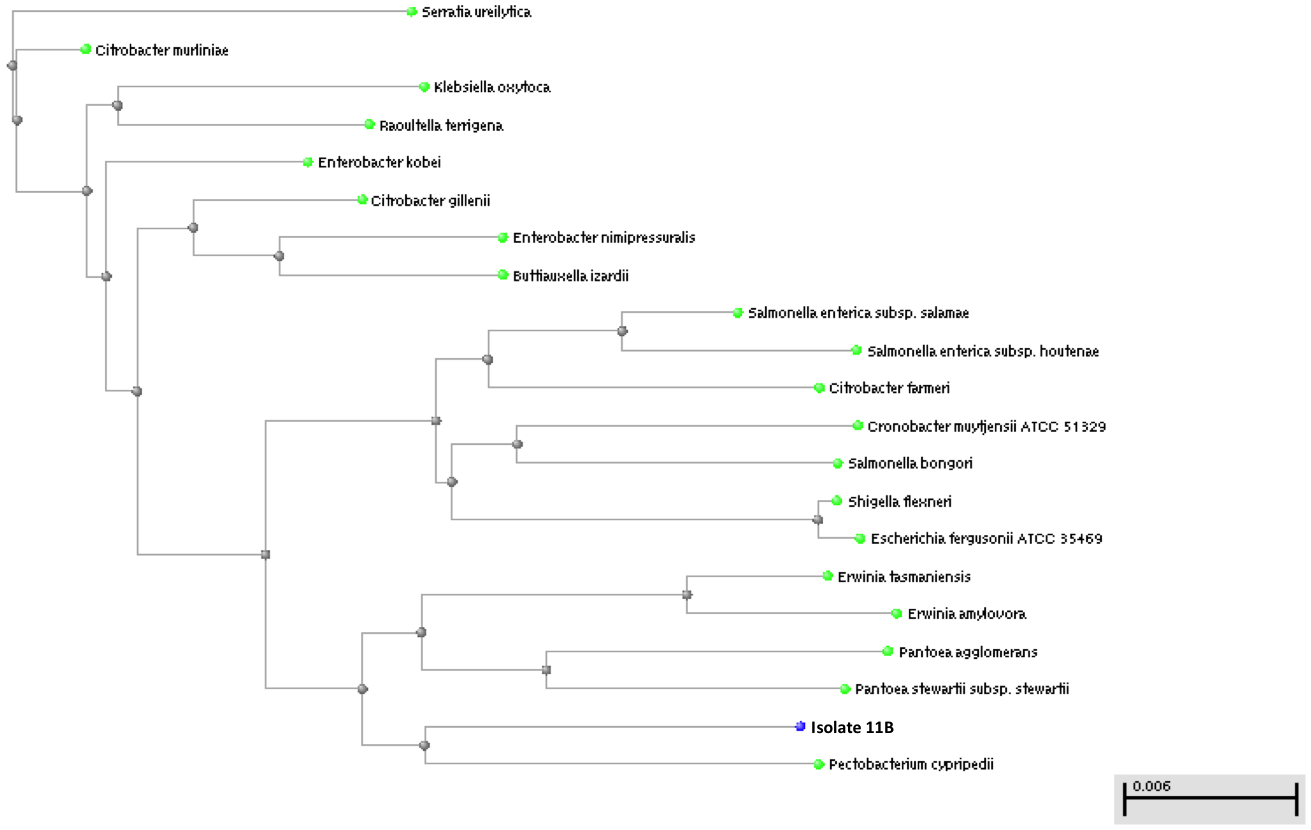


Additional file 7: Phylogenetic tree for the Gram-negative isolate 11B

Supplement: Additional file 7: — Phylogenetic tree for the Gram-negative isolate 11B. (DOCX 140 kb) [file 12866_2015_609_MOESM7_ESM.docx]

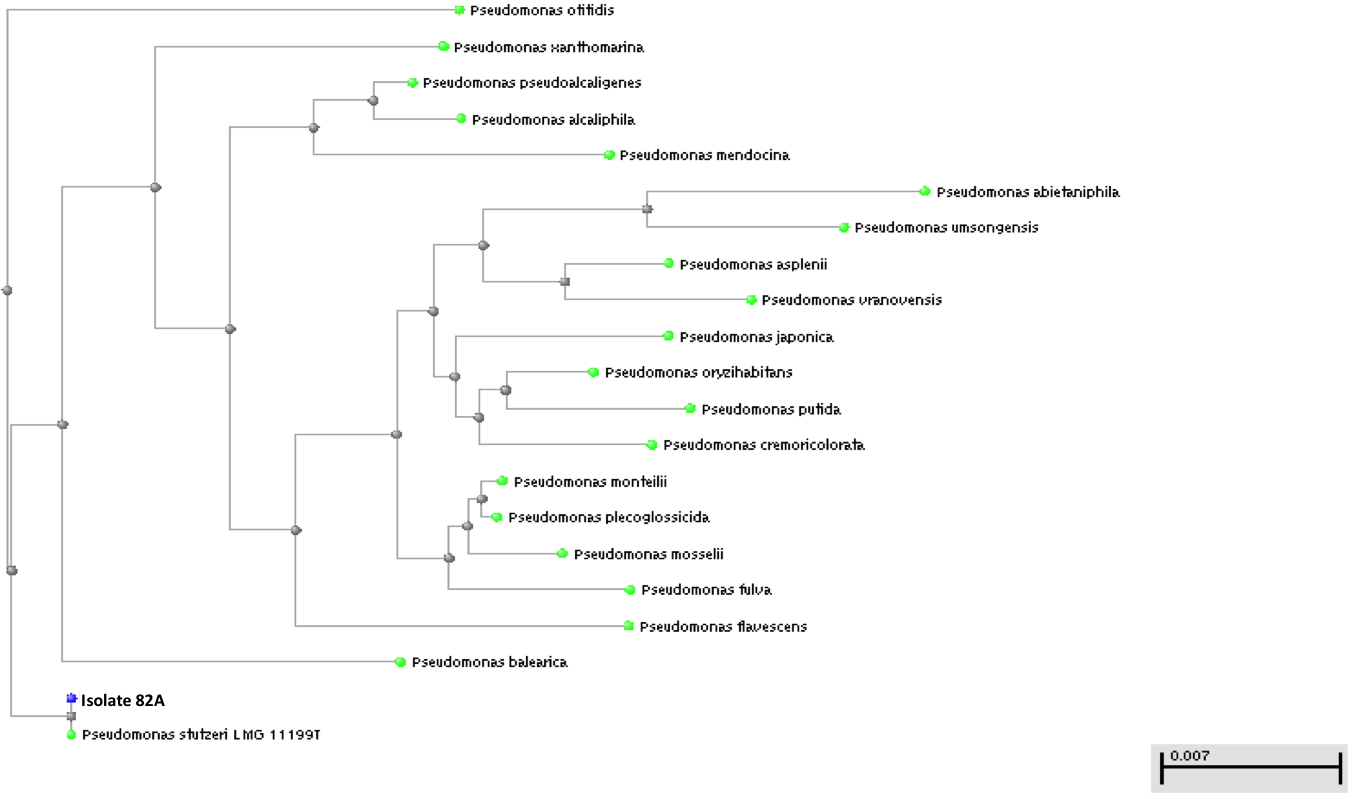


Additional file 8: Phylogenetic tree for the Gram-negative isolate 82A

Supplement: Additional file 8: — Phylogenetic tree for the Gram-negative isolate 82A. (DOCX 142 kb) [file 12866_2015_609_MOESM8_ESM.docx]
